# Impact of COVID-19 Pandemic on Weight and BMI among UK Adults: A Longitudinal Analysis of Data from the HEBECO Study

**DOI:** 10.3390/nu13092911

**Published:** 2021-08-24

**Authors:** Samuel J. Dicken, John J. Mitchell, Jessica Newberry Le Vay, Emma Beard, Dimitra Kale, Aleksandra Herbec, Lion Shahab

**Affiliations:** 1 Department of Behavioural Science and Health, University College London, London WC1E 6BT, UK; john.mitchell.12@ucl.ac.uk (J.J.M.); e.beard@ucl.ac.uk (E.B.); dimitra.kale.09@ucl.ac.uk (D.K.); a.herbec@ucl.ac.uk (A.H.); lion.shahab@ucl.ac.uk (L.S.); 2 Policy and Implementation Research, Cancer Research UK, London E20 1JQ, UK; jessica.newberrylevay@cancer.org.uk; 3 Department of Clinical, Educational and Health Psychology, University College London, London WC1E 6BT, UK

**Keywords:** COVID-19, bodyweight, BMI, health behaviours, diet, weight management

## Abstract

COVID-19-related restrictions impacted weight and weight-related factors during the initial months of the pandemic. However, longitudinal analyses are scarce. An online, longitudinal study was conducted among self-selected UK adults (n = 1818), involving three surveys (May–June, August–September, November–December 2020), covering anthropometric, sociodemographic, COVID-19-related and behavioural measures. Data were analysed using generalised estimating equations. Self-reported average weight/body mass index (BMI) significantly increased between the May–June period and the August–September period (74.95 to 75.33 kg/26.22 kg/m^2^ to 26.36kg/m^2^, *p* < 0.001, respectively), and then significantly decreased to November–December (to 75.06 kg/26.27 kg/m^2^, *p* < 0.01), comparable to May–June levels (*p* = 0.274/0.204). However, there was great interindividual variation, 37.0%/26.7% increased (average 3.64 kg (95% confidence interval: 3.32, 3.97)/1.64 kg/m^2^ (1.49, 1.79)), and 34.5%/26.3% decreased (average 3.59 kg (3.34, 3.85)/1.53 kg/m^2^ (1.42, 1.63)) weight/BMI between May–June and November–December. Weight/BMI increase was significantly negatively associated with initial BMI, and positively associated with monthly high fat, salt and sugar (HFSS) snacks intake and alcohol consumption, and for BMI only, older age. Associations were time-varying; lower initial BMI, higher HFSS snacks intake and high-risk alcohol consumption were associated with maintaining weight/BMI increases between August–September and November–December. The average weight/BMI of UK adults fluctuated between May–June and November–December 2020. However, the substantial interindividual variation in weight/BMI trajectories indicates long-term health impacts from the pandemic, associated with food and alcohol consumption.

## 1. Introduction

Since the start of 2020, the COVID-19 pandemic has seen the introduction of severe lockdown restrictions to limit avoidable morbidity and mortality. In the UK, COVID-19 lockdown restrictions were first imposed on 23 March 2020. Restrictions were then eased from June and July onwards before harsher restrictions returned at the start of October. Various forms of lockdowns were then enforced across the four nations of the UK in November and December 2020. The timing and duration of lockdowns varied across devolved nations. All nations were under lockdown for several weeks during November and December before a tiered restriction system was introduced, where some regions remained under strict lockdown whilst other regions faced more relaxed restrictions.

Obesity-related diseases are a major cause of global morbidity and mortality [1]. Within the UK, the prevalence of overweight and obesity stands at over 60% and continues to rise [2]. Health behaviours including physical activity, diet, smoking and alcohol consumption can impact on bodyweight [3,4,5,6], and such health behaviours have been shown to be affected by COVID-19 lockdown restrictions [7,8,9,10]. Lockdown restrictions can also impact on other individual, social and environmental factors that influence energy intake and expenditure [7,8]. Indeed, reports from the initial months of the pandemic suggest that average weight and body mass index (BMI) have increased significantly by 1.57 kg (95% confidence interval: 1.01–2.14) and 0.31 kg/m^2^ (95%CI: 0.17–0.45), respectively, compared to before the pandemic [11]. However, reports also suggest that despite significant proportions of people increasing weight and BMI (11.2–72.4%), many also decreased in weight and BMI (7.2–51.4%) during the start of the pandemic [11,12,13,14,15,16,17]. These initial weight trajectories during COVID-19 are associated with several sociodemographic, COVID-19-related and behavioural factors including age, gender, initial BMI, pandemic living and working conditions, diet, physical activity and alcohol intake [7,9,10,12,18,19,20,21]. Short-term shifts in health behaviours can result in small, yet meaningful changes in bodyweight, as seen with seasonal holiday weight gain during the winter months, which accounts for a large proportion of annual weight gain [22,23]. If these weight/BMI changes seen during the COVID-19 pandemic persist or continue to shift with changes in lockdown restrictions, these changes in health risk could have long-lasting impacts on population health.

However, studies to date assessing the influence of the COVID-19 pandemic on weight/BMI have largely been cross-sectional and during the initial months of the pandemic [21]. Other reports have shown that a range of health behaviours and weight-related factors have been impacted over longer periods of the pandemic [12,24,25]. Little is known about the weight/BMI trajectories of individuals beyond summer 2020, nor over longer periods of the pandemic. In the UK, no studies to date have compared the strict lockdown conditions of May–June 2020, to the eased restrictions in August–September, and again compared this to the tighter restrictions in November–December 2020. 

As described above, since few studies have looked at longitudinal changes beyond the initial pandemic, considering various drivers of overweight/obesity, this study aims to identify if any weight or BMI change during the COVID-19 pandemic was long-term and has been maintained for 6 months of follow-up, and to identify the predictors of long-term weight or BMI change from baseline to 6-months follow-up. Using a longitudinal sample, this study sought to answer the following research questions:

RQ1. What was the average (i) weight and (ii) BMI in a sample of UK adults at the beginning of the COVID-19 pandemic, and at 3-months and 6-months follow-ups?

RQ2. To what extent were sociodemographic, COVID-19-related and behavioural factors associated with a change in (i) weight and (ii) BMI in a sample of UK adults from the beginning of the COVID-19 pandemic to 6-months follow-up?

## 2. Materials and Methods

### 2.1. Study Design

This study is a longitudinal analysis of data from an ongoing online study of adults, the HEalth BEhaviours during the COVID-19 pandemic (HEBECO) study (https://osf.io/sbgru/). HEBECO is a longitudinal study in the UK assessing the impact of the COVID-19 pandemic on health behaviours, their influences and their outcomes. The study was approved by the Ethics Committee at the UCL Division of Psychology and Language Sciences (CEHP/2020/759). Self-selected participants were recruited through multiple online channels including paid and unpaid advertisements across social media (Facebook, Google and Reddit) and mailing lists of UK universities, charities, local government and networks within Cancer Research UK and Public Health England. The full recruitment strategy is available online (https://osf.io/sbgru/). Participants gave their consent prior to data collection. Data were captured and managed by the REDCap electronic data system at UCL [26,27]. Participants were followed up at 1, 3 and 6 months after their baseline survey via email (except for participants that explicitly opted out), with up to three reminders sent at each follow-up to complete the survey. The surveys used in this analysis span a period of 8 months since the beginning of the pandemic (May to December 2020). The 3-months follow-up survey corresponds to the periods of eased pandemic restrictions during August–September 2020, and the 6-months follow-up survey corresponds to the tighter restrictions during November–December 2020. This analysis uses data from the baseline, 3-months and 6-months follow-up surveys. The study protocol was pre-registered on the Open Science Framework (OSF) before analysis (https://osf.io/pr68k/). Deviations from the pre-registered protocol are described in the Supplementary Materials.

### 2.2. Study Sample

The analysis uses data from UK adults (18+) who completed the baseline survey between 5 May and 14 June 2020 (inclusive) and also provided data of interest at the 6-months follow-up survey as a minimum, for the outcome variables outlined below. 

To enable calculating BMI, the analytical sample for RQ1 and RQ2 includes as a minimum, all participants who self-reported height and weight at the baseline survey, and, at least, also self-reported weight at the 6-months follow-up survey. 

### 2.3. Survey

The survey was developed by a multi-disciplinary team, with support from Cancer Research UK and Public Health England. The survey covered a range of topics including sociodemographics, anthropometrics, physical and mental health, social isolation experienced during the COVID-19 pandemic and health behaviours including physical activity, diet, alcohol consumption and smoking. The baseline survey retrospectively covered the same topics from before the COVID-19 pandemic (i.e., pre-COVID-19). The baseline survey took around 20 minutes, and follow-up surveys around 15 minutes.

Full details of outcome and predictor measures can be found in the Supplementary Materials and on OSF (https://osf.io/pr68k/).

#### 2.3.1. Outcomes

Changes in self-reported weight and BMI were the primary and secondary outcomes of interest, respectively. Self-reported anthropometrics are a reliable measure generally; although, individuals tend to overreport height and underreport weight [28].

Change in self-reported weight: Participants were asked at baseline and at 3- and 6-months follow-up surveys, ‘How much do you weigh? Please try to be as accurate as possible’. Participants could answer using a drop-down box in 1 lb increments (or equivalent metric measure), or answer ‘don’t know’ or ‘prefer not to say’. Weight was analysed in kg as a continuous variable for both RQ1 and RQ2. 

Change in self-reported BMI: At baseline, participants were asked ‘What is your height? Please try to be as accurate as possible’. Participants could answer using a drop-down box in 1 inch increments (or equivalent metric measure), or answer ‘don’t know’ or ‘prefer not to say’. BMI was calculated as weight (kg)/squared height (m^2^), with height converted into metres. BMI was analysed as a continuous variable for both RQ1 and RQ2. In the sensitivity analysis, BMI was also analysed as a categorical variable using World Health Organisation (WHO) BMI categories [29,30].

For RQ2, we computed continuous change scores in self-reported weight and BMI from baseline to 3-months follow-up and baseline to 6-months follow-up surveys. This produced two change scores each for weight and BMI at 3 months and 6 months, which were combined to form two outcome variables, ‘change in self-reported weight’ and ‘change in self-reported BMI’.

#### 2.3.2. Explanatory Variables

Baseline sociodemographic variables included gender, BMI, age, ethnicity, occupation and work from home and a socioeconomic score based on self-reported income, housing tenure and education. COVID-19-related variables included baseline living arrangements, isolation status reported at each timepoint and quality of life, based on self-reported ratings of quality of living, wellbeing, social and family relationships, also reported at each timepoint. Time-invariant behavioural variables included high fat, salt and sugar (HFSS), ΔHFSS snacks, ΔHFSS meals and Δfruit and vegetable intake change scores, derived from pre-pandemic dietary intakes retrospectively reported in the baseline survey being deducted from dietary intakes reported at the time of the baseline survey (i.e., at the start of the pandemic), as initial dietary changes have been associated with maintaining pandemic weight gain [12]. The time-varying behavioural variables included HFSS snacks intake, HFSS meals intake, fruit and vegetables intake, physical activity (given the link between reduced physical activity and pandemic weight gain [7,10,21], based on WHO weekly physical activity recommendations of two days per week for strengthening physical activity, and 150 minutes per week for aerobic physical activity [31]), alcohol consumption (based on government low-risk drinking recommendations [32]) and smoking status. HFSS foods intake, physical activity and alcohol consumption were assessed at each wave using previously validated measures (see Supplementary Materials). The food item questions are based on previous research study survey questions and derived from Public Health England’s sugar reduction programme definitions as measures relevant for informing health policy [33,34,35]. The HFSS food items included HFSS meals (ready meals, fast food and takeaways) and HFSS snacks (sugary or sweetened drinks, sweets or chocolate, cakes and biscuits, desserts and savoury snacks).

### 2.4. Statistical Analysis

Statistical analysis was conducted in SPSS Statistics version 27 (IBM). Significance was defined as *p* < 0.05. The statistical analysis plan was pre-registered on OSF prior to analysis (https://osf.io/pr68k/).

We report cross-sectional baseline participant characteristics in RQ1 and unweighted means with 95% confidence intervals (95% CI) for (i) weight and (ii) BMI at each timepoint. We also report the percentage of the sample increasing/decreasing (i) weight or (ii) BMI between timepoints. An increase or decrease was defined as more than a 0.5 kg or 0.5 kg/m^2^ change, respectively, from the reference timepoint (baseline or 3-months follow-up survey), as these are previously used cut-offs [22,23,36]. Lastly, we reported the mean change (with 95%CI) in (i) weight and (ii) BMI in those increasing/decreasing weight/BMI between timepoints.

We used an unadjusted GEE model with identity link function and AR(1) covariance structure (given that participant measures at closer timepoints are expected to be more correlated [37]) to examine changes in self-reported (i) weight and (ii) BMI across 6 months of follow-up during the COVID-19 pandemic. Pairwise time comparisons were conducted between baseline, 3- and 6-months timepoints and inserted into the GEE model as factors. Multiple comparisons were adjusted for using sequential Šidák correction.

GEE models were used to determine the association between the sociodemographic, COVID-19-related and behavioural predictor variables listed above and change in (i) weight and (ii) BMI across timepoints. Time*explanatory variable interactions were inserted into GEE models to assess temporal differences in the association of explanatory variables with continuous changes in (i) weight and (ii) BMI over time. The time variable was categorical, as the trajectory of change in weight and BMI was not expected to be linear [38].

An unadjusted GEE model was computed showing the association between each individual explanatory variable and the change in (i) weight or (ii) BMI across timepoints. Each individual explanatory variable model was then adjusted for a main effect of time and for a time*explanatory variable interaction. A fully adjusted GEE model containing all explanatory variables was then computed. The fully adjusted GEE model was assessed for goodness of fit using Quasi-Likelihood under Independence Model Criterion (QIC) [39]. Then, a fully adjusted GEE model containing all explanatory variables as well as all significant time*explanatory variable interactions from the univariate models with time interactions was computed. Time*explanatory variable interactions were retained in the full GEE model if they improved QIC > 2 over the full GEE model without interactions, and the interaction itself remained significant (*p* < 0.05).

Independent variables were retained after checking for collinearity using Pearson correlations (all correlations *r* < 0.4).

The sensitivity analyses use complete cases only; those participants who self-report weight measures at all three timepoints and height at the baseline survey. Further analyses were also conducted using two binary logistic GEE models with logit link function for an ‘increase’ in weight/BMI vs. ‘all other’, and a ‘decrease’ in weight/BMI vs. ‘all other’, with a minimum 5% weight/BMI change from baseline cut-off to define an increase or decrease in weight/BMI. Such magnitude of weight change has been used to define clinically meaningful weight loss [40,41].

For BMI only, analyses were stratified by WHO BMI categories [30], i.e., associations with an ‘increased BMI’, vs. ‘all other’ were assessed in individuals with a baseline BMI < 25 kg/m^2^ (normal weight and underweight), and associations with a ‘decreased BMI’ vs. ‘all other’ in individuals with a baseline BMI ≥ 25 kg/m^2^ (overweight and obesity).

Further details of this analysis are provided in the Supplementary Materials.

Bayes Factor analyses in the event of non-significant findings were pre-registered online for physical activity, BMI, snacking, alcohol consumption, age and gender (https://osf.io/pr68k/), as current literature suggests that reduced physical activity, higher initial BMI, increased snacking, high alcohol consumption, younger age and female gender are associated with weight/BMI gain at the start of the pandemic [7,8,10,21]. Effect sizes were obtained from mean differences in weight change reported in the COVID-19 literature [42]. Alternative hypotheses were modelled using a half-normal distribution with a peak at zero, given that smaller effect sizes nearer to the null are more likely than larger effect sizes [42,43]. The standard deviation (SD) was set to the expected mean difference in weight change reported in the COVID-19 literature for gender and physical activity: a prior mean difference between males and females of 0.20 kg weight change during the pandemic [10] and a prior mean difference between maintaining physical activity and reduced physical activity of 0.23 kg weight change during the pandemic [10]. Bayes factors were then calculated using an online calculator: http://bayesfactor.info/.

## 3. Results

### 3.1. Participant Characteristics

Out of a total of 2992 UK participants over the age of 18 recruited into the HEBECO baseline survey, 1818 (weighted = 1631) participants met the inclusion criteria for analyses. Table 1 shows the unweighted baseline characteristics for the total, included and excluded samples (for weighted participant characteristics, see Supplementary Table S1). There were some differences between included and excluded samples. Included participants were more likely to be female, older, of white ethnicity, under stricter lockdown conditions, a non-smoker, unemployed (which includes retired persons and full-time parents/carers) and to have a higher baseline BMI, a higher quality of life score and higher socioeconomic score, and consumed more portions of fruit and vegetables and fewer portions of HFSS meals at baseline. 

### 3.2. Average (i) Weight and (ii) BMI in UK Adults at the Beginning of the COVID-19 Pandemic and at 3- and 6-Month Follow-Ups during the COVID-19 Pandemic

**Figure 1:**
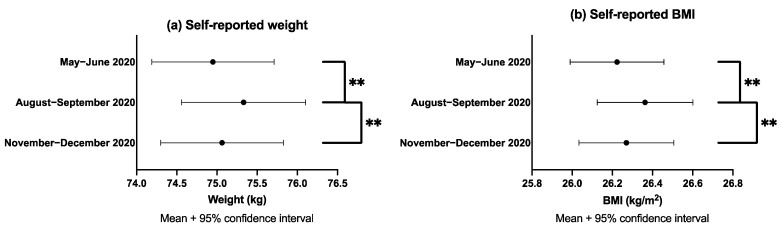
Means and 95% confidence intervals with pairwise comparisons between baseline (May–June 2020), 3-months (August–September 2020) and 6-months follow-up (November–December 2020) for (**a**) weight and (**b**) BMI. ** Denotes pairwise comparisons between timepoints were significant at the 0.005 level.

Weight and BMI both significantly increased from baseline (May–June 2020) to 3-months follow-up (August–September 2020) (74.95 to 75.33 kg, 26.22 to 26.36 kg/m^2^, respectively, both *p* < 0.001) (Figure 1). Weight and BMI then significantly decreased from 3-months follow-up to 6-months follow-up (November–December 2020) (75.33 to 75.06 kg, 26.36 to 26.27 kg/m^2^, respectively, both *p* = 0.003). Weight and BMI did not significantly differ from baseline to 6-months follow-up (74.95 to 75.06 kg, 26.22 to 26.27 kg/m^2^, respectively, *p* = 0.274/0.204). Complete case analysis did not materially alter these findings (Supplementary Tables S2 and S3).

From baseline, 37.0%/26.7% reported an increase in weight/BMI greater than 0.5 kg/0.5 kg/m^2^, and 34.5%/26.3% reported a decrease in weight/BMI at the 6-months follow-up survey, compared to the baseline survey (Table 2). Individuals’ increasing weight/BMI increased on average by 3.64 kg [95%CI: 3.32, 3.97]/1.64 kg/m^2^ [1.49, 1.79]. Individuals decreasing weight/BMI decreased on average by 3.59 kg [3.34, 3.85]/1.53 kg/m^2^ [1.42, 1.63].

### 3.3. Sociodemographic, COVID-19-Related and Behavioural Factors Associated with Changes in (i) Weight and (ii) BMI in UK Adults from the Beginning of the COVID-19 Pandemic to 6-Months Follow-Up

In the unadjusted GEE models (Supplementary Table S4), lower baseline BMI, higher HFSS snacks intake, lower fruit and vegetables intake, and a positive (increased intake) ΔHFSS snacks change score were significantly associated with an increase in self-reported weight/BMI. In the fully adjusted GEE model (Table 3), increases in BMI only were associated with older age (B: 0.005 kg/m^2^ [<0.001, 0.010]). Baseline BMI and HFSS snacks intake remained significantly associated with a change in self-reported weight/BMI, with high-risk alcohol consumption now also significantly associated with an increase in self-reported weight/BMI and neither ΔHFSS snacks change score nor lower fruit and vegetables intake remaining associated. However, based on significant time*explanatory variable interactions from the univariate GEE models (Supplementary Table S4) carried over to the fully adjusted GEE model (Table 3), baseline BMI, HFSS snacks intake and alcohol consumption all demonstrated significant interactions with time and improved model fit. Main effects were not materially altered by the addition of time interactions.

**Figure 2:**
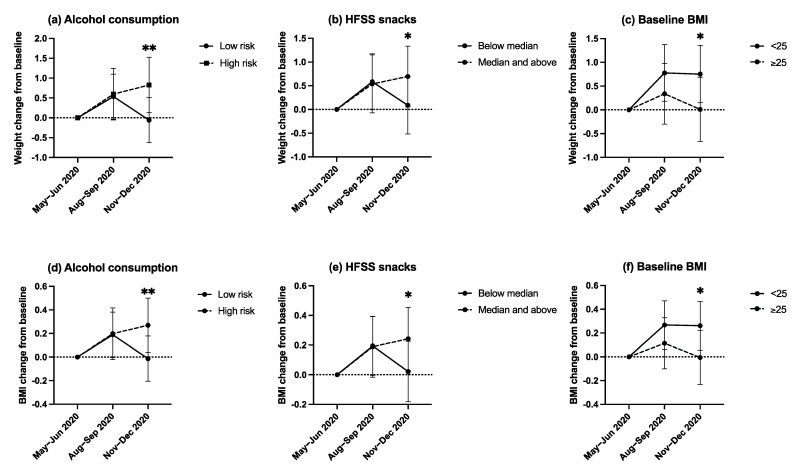
Graphical representations of the time-varying associations of alcohol consumption (**a**), HFSS snacks intake (**b**) and baseline BMI (**c**) with weight change at 3-months (August–September 2020) and 6-months follow-up (November–December 2020). Graphical representations of the time-varying associations of alcohol consumption (**d**), HFSS snacks intake (**e**) and baseline BMI (**f**) with BMI change at 3-months (August–September 2020) and 6-months follow-up (November–December 2020). * Denotes pairwise comparisons between categories were significant at the 0.05 level. ** Denotes pairwise comparisons between categories were significant at the 0.005 level. Median HFSS snacks intake was 34.5 and 40.0 portions per month at 3-months (August–September 2020) and 6-months follow-up (November–December 2020), respectively.

Figure 2 schematically demonstrates the time-varying trends of baseline BMI, HFSS snacks intake and alcohol consumption on change in weight/BMI, using a binary cut-off for baseline BMI (<25 vs. ≥25) and HFSS snacks intake (below median intake vs. median intake and above). The mean weight/BMI change associated with baseline BMI, HFSS snacks intake and alcohol consumption was significantly different at 6-months (November–December 2020) compared to 3-months follow-up (August–September 2020) (all time interactions *p* < 0.001). The association of lower baseline BMI, higher HFSS snacks intake and high-risk alcohol consumption with an increase in weight/BMI was greater at 6-months follow-up than at 3-months follow-up. Pairwise comparisons demonstrated that the change in weight/BMI significantly differed between the binary categories (low-risk vs. high-risk alcohol consumption, <25 vs. ≥25 BMI, below median vs. median and above HFSS snacks intake), at 6- but not at 3-months follow-up. 

### 3.4. Sensitivity Analyses

Unweighted and weighted proportions of BMI categories using complete cases are presented in Supplementary Table S5.

In complete case analysis (Supplementary Tables S6 and S7), baseline BMI and HFSS snacks intake remained significantly associated with a change in weight/BMI. High-risk alcohol consumption became non-significantly associated with an increase in weight/BMI and lower fruit and vegetable intake became significantly associated with an increase in weight/BMI. Age was no longer significantly associated with a change in BMI. Similarly, for changes in weight/BMI, baseline BMI, HFSS snacks intake and alcohol consumption interactions with time also remained significant and improved model fit, though main effects were altered slightly (Supplementary Table S7).

In the binary outcome analyses (Supplementary Tables S8–S11), weight increase was associated with younger age and lower fruit and vegetable intake. Similarly, BMI increase from a BMI < 25 was associated with a lower baseline BMI and lower fruit and vegetable intake. Weight decrease was associated with non-female gender, a higher baseline BMI, lower HFSS snacks intake and low-risk alcohol consumption (≤14 weekly units). Likewise, BMI decrease from a BMI ≥ 25 was associated with non-female gender, younger age, lower HFSS snacks intake, higher fruit and vegetable intake and low-risk alcohol consumption (≤14 weekly units). No time*explanatory variable interactions significantly improved the fully adjusted GEE models for the binary weight outcomes (Supplementary Table S9). There were no significant time*explanatory variable interactions for the binary BMI outcomes (Supplementary Tables S10 and S11). 

The Bayes Factors (BF) suggest the data were insensitive to detect an effect of gender (BF = 2.51) or physical activity (BF = 0.60) on change in weight.

## 4. Discussion

In this sample of UK adults, fluctuations in self-reported weight/BMI were seen during the COVID-19 pandemic between May and December 2020. The average changes, however, do not reflect the considerable interindividual variability in weight/BMI change. Over half of participants self-reported a change in their weight/BMI at their 6-month follow-up survey compared to their baseline survey at the start of the pandemic. Taken together, our results suggest that diet and alcohol consumption during the pandemic are associated with longer-term changes in weight/BMI. These factors relate to maintaining the initial weight/BMI gain, which is masked by the return to baseline levels, as seen in the population at large.

A systematic review and meta-analysis also demonstrated an overall trend for a small, significant increase in population mean weight (1.57 kg) and BMI (0.31 kg/m^2^) during the initial months of the pandemic [11,12]. A longitudinal study in the US showed that average weight/BMI was still significantly increased above peak lockdown levels (April–May 2020) by September–October 2020 [12]. However, many reports during COVID-19 show that despite a small increase in population weight/BMI, many individuals experienced a reduction in weight/BMI [9,10,13,14,15,16,17]. The average weight increase/decrease reported in this study are larger than the 0.6–3.0 kg/2.0–2.9 kg average weight increase/decrease reported in a systematic review of COVID-19 studies up to July 2020 [7]. 

Weight changes can be beneficial or detrimental to health and wellbeing. Understanding the factors associated with these changes is crucial to develop targeted interventions. Increased HFSS food intake, snacking and alcohol consumption have all been previously identified as important predictors of initial pandemic weight/BMI gain [7,9,10,12,18,19,20,21,25]. Studies in the UK and internationally report large interindividual variability in dietary changes as a result of the COVID-19 pandemic [44], which in turn have been associated with weight change [7,12,14]. Sustained high intakes of ultra-processed, HFSS foods beyond peak lockdown have been associated with maintaining any weight gained during the start of the pandemic [12]. Previous studies have also identified changes in physical activity as an important predictor of weight change [7,9,10,12,18,19,20,21], and having an overweight or obese BMI or being female as important predictors of initial pandemic weight gain [7,10,14,45]. In our study, a reduction in physical activity was not significantly associated with a change in weight/BMI, initial BMI was inversely associated with weight/BMI gain, and gender was not significantly associated with a change in weight/BMI in the main analyses between May and December 2020. However, being female was associated with lower odds of decreasing weight/BMI in the sensitivity analyses. Bayes Factor analysis demonstrated that the data in this study was insensitive to detect an effect of gender or physical activity on weight change. One of the few cross-sectional studies beyond the initial months during October 2020 also found no impact of gender on weight gain [25].

The pandemic and its restrictions have resulted in lifestyle shifts for many individuals, ranging from minor to major changes. The early stages of the pandemic have been compared to the holiday season, where short-term shifts in lifestyle can impact on long-term weight management [22]. The pandemic may have resulted in behavioural changes due to social restrictions imposed, changes to working patterns, or from COVID-19-related stress and the coping strategy used [46]. However, whether individuals adaptively coped (e.g., through physical activity or fruit and vegetable intake) or maladaptively coped (e.g., through alcohol consumption or HFSS snacking) differs greatly between individuals within studies [47], with maladaptive strategies being linked to pandemic weight gain [12,46]. Individuals may have used maladaptive coping strategies such as increased snacking or alcohol consumption at the start of pandemic, but then sustained these behaviours through habit formation, subsequently maintaining any initial weight/BMI increase after lockdown restrictions were eased.

Dietary changes are important determinants of weight change as brief periods of weight gain are typically driven by energy overconsumption, such as from increased ultra-processed, HFSS food intake [22,45,48,49]. Studies have noted increased snacking alongside elevated stress, appetite, boredom, low craving control and higher emotional eating during the pandemic [12,46,50]. Given the high palatability of HFSS snacks, individuals may have demonstrated emotional eating behaviours and consumed additional HFSS snack foods as a comfort mechanism to deal with COVID-19 related stress, depression or anxiety [50,51]. HFSS foods also tend to have long shelf lives, be cheaper and more readily accessible. Individuals may have ‘stocked-up’ on HFSS foods through less frequent shopping trips [50]. Being stuck at home may have increased sedentary time and time spent watching TV, further contributing to increased snacking [52]. Initial shifts in HFSS snacking behaviours may then have been maintained through habit formation, increasing the risk of energy overconsumption and weight gain.

Alcohol consumption may also have been used as a maladaptive coping mechanism for COVID-19-related stress, boredom and depression [53,54]. Consumption may have increased through both a greater frequency of binge-drinking and frequency of consumption. High-risk alcohol consumption has been associated with unfavourable dietary changes during the pandemic [53], possibly by influencing appetite and food choice, thus increasing energy intake both directly and indirectly [52,54]. Alcohol consumption may also have shifted from the closures of bars and pubs, with more purchases made for home consumption [8].

Reduced physical activity may not have resulted in weight/BMI change in this study from compensatory behaviours; those exercising more may also have consumed more food, and those exercising less may have consumed less food. There is also a complex relationship between physical activity and alcohol consumption; individuals may have attempted to compensate for high-risk alcohol consumption with additional physical activity to offset energy intake [55,56,57]. 

The lack of association between female gender and weight/BMI gain in this study may reflect the coping strategies of female participants, and the complex gender differences in changes in health behaviours during the pandemic. Some studies find women increasing exercise more than men [58], being less likely to alter their alcohol intake [55] and being more likely to increase fruit and vegetables intake, eat less or partake in healthy eating [8,21,58]. However, UK women have also been shown to eat both more and less during the pandemic [18]. Greater COVID-19-related stress was reported in females during the start of the pandemic, but psychological distress tended to return to pre-pandemic levels by mid-2020 [59,60]. Further, the impact of COVID-19-related stress on weight/BMI management likely depends on the coping strategies employed, with women in the UK being more likely to use any coping strategy compared to men [47]. 

Prior to the pandemic, around two thirds of UK adults were living with overweight or obesity, increasing year on year [2]. The results of this study suggest that the pandemic is associated with longer-term changes in weight/BMI, which could contribute to the existing obesity epidemic. Considering that obesity alongside adverse dietary choices and alcohol consumption is one of the leading causes of disability-adjusted life years and years of life lost in the UK [61,62], there is greater urgency than ever to support people to make healthy behavioural choices to support weight management.

The association between high HFSS snacks consumption and weight/BMI gain highlights the need for policy action regarding diet. Greater effort needs to be made to increase the accessibility and availability of healthy dietary choices, and not just limiting the accessibility of unhealthful alternatives. 

If maladaptive strategies to stress are resulting in changes in health behaviours that impact on weight management, then greater support for encouraging adaptive coping strategies should be considered. For example, providing resources for individuals to develop coping strategies towards benefitting from the positive mental affect of physical activity, practicing mindfulness, breathing or encouraging sufficient sleep [52]. The association here between high-risk drinking and maintained weight gain indicates that there needs to be additional work to screen for individuals with high-risk alcohol consumption, to advise on how to avoid using alcohol to deal with stress and anxiety [54] and to provide adequate treatment to support a reduction in alcohol intake [63].

This study has several strengths. This is the first UK study examining weight change across the first year of the COVID-19 pandemic from May to December 2020. This longitudinal study builds and expands upon the largely cross-sectional literature published to date, providing a greater understanding of the long-term impacts of the pandemic on weight management. The analysis included a range of variables that reflect the wide-ranging impact of the pandemic (including sociodemographics, lifestyle, wellbeing and health behaviours), and with time-varying measures to reflect the changing conditions of the pandemic over time. An important further contribution of this study is that it assessed the associations of a range of health behaviours relevant to weight management (physical activity, diet, alcohol and smoking). The use of GEE modelling for the longitudinal analysis provided several advantages over typical analytical methods. Finally, the use of both weight and BMI as outcome measures, and complete case analyses and sensitivity analyses with binary cut-offs demonstrating largely consistent associations of baseline BMI, HFSS snacks intake and alcohol consumption with weight/BMI management, indicates the robustness of these findings.

However, several limitations may have introduced bias. First, the study sample was self-selected and featured a predominantly female, younger, well-educated cohort. Second, there were differences in ethnic diversity between the included and excluded samples, and data were unweighted for the longitudinal analyses. All of which may limit the generalisability of the findings to the UK population. Third, due to the observational nature of this study, causality cannot be determined. Fourth, as is the case for the majority of epidemiological studies during the COVID-19 pandemic [11], measures of interest were self-reported, including for weight and height that may be underreported and overreported, respectively [28]. It was also not possible to determine whether and how participants measured their height and weight. However, change scores as outcome variables with participants acting as their own controls helped to minimise within-subject measurement error, though it cannot be guaranteed that participants used the same measurement methods across time. In addition, participants were not explicitly asked if their weight had changed, nor told that weight change was an outcome of interest, reducing the risk of expectation bias. Previous analyses have shown that longitudinal repeat measures of weight and height over time in older UK adults are highly stable over years of follow-up [64]. While the use of BMI as an outcome carries several limitations [65], at a population level, there is a reliable association with long-term health risk [66]. Fifth, the study did not consider body composition, which could provide further insights into the weight-related health impacts of the pandemic. Sixth, previous studies have compared weight/BMI to pre-pandemic levels, whereas this study compared weight/BMI to baseline levels at the start of the pandemic. Although the baseline survey happened at the beginning of imposed restrictions, given the large weight change from pre- to during-pandemic [11], this may have underestimated the changes in weight/BMI in this study. Lastly, participants were asked about their behaviours in the past week or month, which may have introduced a recall bias. 

## 5. Conclusions

In this sample of UK adults, average self-reported weight/BMI fluctuated across the first year of the pandemic but was not significantly different in November–December compared with May–June 2020. However, this masks wide interindividual variability in weight/BMI change. Older age was associated with an increase in BMI. Baseline BMI, HFSS snacks intake and alcohol consumption showed time-varying associations with increasing weight/BMI, being associated with maintenance of initial pandemic weight gain through to the latter part of 2020. These findings highlight the long-term health impacts of the pandemic on weight change, to guide health policy and direct attention to those at increased risk of likely poorer health outcomes associated with weight gain.

## Figures and Tables

**Table 1:** Unweighted baseline participant characteristics for the total, included and excluded samples.

	Total Sample	Included Sample	Excluded Sample	
	Unweighted (%)	Unweighted (%)	Unweighted (%)	*p*
N	2992	1818	1174	
Gender				0.107
All other	31.4%	30.3%	33.0%	
Female	68.6%	69.7%	67.0%	
Ethnicity				**<0.001**
All other	6.3%	4.5%	9.1%	
White	93.7%	95.5%	90.9%	
Mean BMI [SD] (N = 2783)	26.1 [5.2]	26.2 [5.1]	25.7 [5.3]	**0.018**
Mean Age [SD]	47.9 [15.5]	51.7 [14.3]	42.0 [15.4]	**<0.001**
Occupation and work from home (N = 2790)				**<0.001**
Unemployed (including retired persons and full-time parents/carers)	28.6%	33.2%	21.5%	
Employed working from home	51.6%	48.0%	57.1%	
Employed not working from home	19.8%	18.8%	21.3%	
Socioeconomic score				**<0.001**
Income < GBP 50 k, unowned housing and no higher education	4.9%	4.1%	6.1%	
1 of: ≥GBP 50 k income, housing ownership/mortgage or higher education	27.8%	24.2%	33.3%	
2 of: ≥GBP 50 k income, housing ownership/mortgage or higher education	38.5%	40.7%	35.0%	
All of: ≥GBP 50 k income, housing ownership/mortgage and higher education	28.8%	31.0%	25.5%	
Living conditions				**<0.001**
Alone	16.8%	16.8%	16.9%	
With children (with or without adults)	19.5%	17.1%	23.3%	
With adults only	63.6%	66.1%	59.8%	
Isolation status (N = 2946)				0.285
Total or some isolation	79.3%	79.9%	78.3%	
General or no isolation	20.7%	20.1%	21.7%	
Mean Quality of Life [SD] (1–5) (N = 2889)	3.4 [0.8]	3.4 [0.8]	3.3 [0.8]	**<0.001**
Mean HFSS snacks (portions per month) [SD] (N = 2609}	58.4 [45.2]	56.8 [44.1]	61.4 [47.0]	**0.012**
Mean HFSS meals (portions per month) [SD] (N = 2618)	6.6 [8.3]	5.8 [6.6]	8.0 [10.6]	**<0.001**
Mean Fruit and vegetables (portions per month) [SD] (N = 2647)	44.0 [18.0]	45.4 [17.1]	41.4 [19.3]	**<0.001**
Mean ΔHFSS snacks change score (portions per month) [SD] (N = 2609)	8.8 [34.5]	9.4 [33.3]	7.9 [36.5]	0.300
Mean ΔHFSS meals change score (portions per month) [SD] (N = 2618)	−1.4 [7.9]	−1.3 [7.1]	−1.5 [9.0]	0.462
Mean ΔFruit and vegetables change score (portions per month) [SD] (N = 2647)	−0.1 [12.5]	0.02 [11.6]	−0.4 [13.9]	0.377
Physical activity (N = 2825)				**0.002**
All other	72.5%	70.6%	75.8%	
Reduced	27.5%	29.4%	24.2%	
Alcohol consumption (N = 2772)				0.996
≤14 weekly units	81.0%	81.0%	80.9%	
>14 weekly units	19.0%	19.0%	19.1%	
Smoking status				**<0.001**
Yes	18.6%	14.0%	25.7%	
No	81.4%	86.0%	74.3%	

*p*-values are for comparisons between the analytical and excluded samples. 133 (weighted = 141) participants were excluded due to reporting ‘don’t know’ or ‘prefer not to say’ to the height or weight questions at the baseline or 6-months follow-up surveys. Bold indicates statistical significance. SD: Standard Deviation.

**Table 2:** Unweighted proportions increasing or decreasing weight/BMI, and mean changes in proportions increasing or decreasing weight/BMI with 95% confidence intervals.

		Self-Reported Weight				Self-Reported BMI			
		Increase		Decrease		Increase		Decrease	
	N	%	Mean [95% CI]	%	Mean [95% CI]	%	Mean [95% CI]	%	Mean [95% CI]
Baseline–3 mo.	1543	36.9	3.23 [2.91, 3.55]	27.7	−2.99 [−3.25, −2.72]	25.9	1.47 [1.32, 1.62]	20.2	−1.28 [−1.40, −1.17]
3 mo.–6 mo.	1543	27.9	2.81 [2.50, 3.13]	35.4	−3.10 [−3.29, −2.81]	17.4	1.35 [1.19, 1.52]	23.7	−1.40 [−1.51, −1.30]
Baseline–6 mo.	1818	37.0	3.64 [3.32, 3.97]	34.5	−3.59 [−3.85, −3.34]	26.7	1.64 [1.49, 1.79]	26.3	−1.53 [−1.63, −1.42]

An increase or decrease in weight/BMI defined as an increase or decrease greater than 0.5 kg/0.5 kg/m^2^ compared to the reference time point. CI: Confidence Interval. Mo: months.

**Table 3:** Full GEE model containing all predictor variables and the full GEE model containing all predictor variables and significant predictor*time interactions from univariate models adjusted for time.

	**Change in Self-Reported Weight QIC = 47,855.838**				**Change in Self-Reported BMI QIC = 5903.284**			
**All predictors (N = 1640)**	**W χ^2^**	***p***	**B [95% CI]**	**SE**	**W χ^2^**	***p***	**B [95% CI]**	**SE**
Gender	3.000	0.083			3.678	0.055		
All other			Reference				Reference	
Female			0.400 [−0.053, 0.853]	0.2310			0.144 [−0.003, 0.291]	0.0750
Ethnicity	<0.001	0.988			0.004	0.952		
All other			Reference				Reference	
White			0.050 [−0.605, 0.614]	0.3108			0.007 [-0.221, 0.225]	0.1164
Baseline BMI	**12.985**	**<0.001**	**−0.095 [−0.147, −0.044]**	**0.0265**	**12.883**	**<0.001**	**−0.034 [−0.052, −0.015]**	**0.0094**
Age	3.519	0.061	0.014 [−0.001, 0.028]	0.0073	**3.866**	**0.049**	**0.005 [<0.001, 0.010]**	**0.0025**
Occupation and work from home	0.101	0.951			0.184	0..912		
Unemployed			Reference				Reference	
Employed working from home			0.079 [−0.438, 0.597]	0.2641			0.038 [−0.144, 0.221]	0.0932
Employed not working from home			0.035 [−0.594, 0.663]	0.3207			0.018 [−0.200, 0.235]	0.1112
Socioeconomic score	4.521	0.210			5.210	0.157		
Income <GBP 50 K, unowned housing and no higher education			Reference				Reference	
1 of: ≥GBP 50 k income, housing ownership/mortgage or higher education			−0.003 [−1.420, 1.414]	0.7229			0.011 [−0.458, 0.481]	0.2394
2 of: ≥GBP 50 k income, housing ownership/mortgage or higher education			−0.354 [−1.730, 1.023]	0.7024			−0.132 [−0.586, 0.322]	0.2316
All of: ≥GBP 50 k income, housing ownership/mortgage and higher education			−0.595 [−1.967, 0.778]	0.7004			−0.215 [−0.669, 0.238]	0.2314
Living conditions	0.109	0.947			0.091	0.955		
Alone			Reference				Reference	
With children (with or without adults)			0.088 [−0.559, 0.734]	0.3298			0.032 [−0.188, 0.253]	0.1126
With adults only			0.013 [−0.502, 0.529]	0.2630			0.012 [−0.166, 0.191]	0.0911
Isolation status	0.889	0.346			0.907	0.341		
Total or some isolation			Reference				Reference	
General or no isolation			−0.144 [−0.442, 0.155]	0.1523			−0.051 [−0.156, 0.054]	0.0536
Quality of Life	0.344	0.557	0.063 [−0.147, 0.273]	0.1072	0.477	0.490	0.027 [−0.049, 0.102]	0.0386
HFSS snacks intake	**15.056**	**<0.001**	**0.010 [0.005, 0.015]**	**0.0026**	**15.683**	**<0.001**	**0.004 [0.002, 0.005]**	**0.0009**
HFSS meals intake	1.912	0.167	0.016 [−0.006, 0.038]	0.0113	1.548	0.213	0.005 [−0.003, 0.012]	0.0038
Fruit and vegetables intake	3.757	0.053	−0.009 [−0.018, 0.001]	0.0047	3.719	0.054	−0.003 [−0.006, 0.000]	0.0017
HFSS snacks change score	2.874	0.090	0.009 [−0.001, 0.018]	0.0050	3.199	0.074	0.003 [0.000, 0.006]	0.0016
HFSS meals change score	0.135	0.713	−0.005 [−0.033, 0.023]	0.0142	0.131	0.717	−0.002 [−0.011, 0.008]	0.0048
Fruit and vegetables change score	0.486	0.486	−0.006 [−0.023, 0.011]	0.0088	0.604	0.437	−0.002 [−0.008, 0.004]	0.0030
Physical activity	0.019	0.890			0.010	0.922		
All other			Reference				Reference	
Reduced			0.021 [−0.276, 0.318]	0.1517			−0.005 [−0.110, 0.100]	0.0535
Alcohol consumption	**6.243**	**0.012**			**5.557**	**0.018**		
≤14 weekly units			**Reference**				**Reference**	
>14 weekly units			**0.496 [0.107, 0.885]**	**0.1985**			**0.153 [0.026, 0.281]**	**0.0651**
Smoking status	0.020	0.887			0.012	0.911		
Yes			Reference				Reference	
No			0.042 [−0.531, 0.614]	0.2920			0.011 [−0.190, 0.213]	0.1026
	**Change in self-reported weight QIC = 47,599.355**				**Change in self-reported BMI QIC = 5874.771**			
**All predictors + significant time interactions (N = 1640)**	**W χ^2^**	***p***			**W χ^2^**	***p***		
Time*Baseline BMI	**13.675**	**<0.001**			**12.937**	**<0.001**		
Time*HFSS snacks intake	**17.525**	**<0.001**			**16.311**	**<0.001**		
Time*Alcohol consumption	**14.437**	**<0.001**			**14.006**	**<0.001**		

Models also included Time as a covariate. For ‘All predictors + significant time interactions’, Type III tests for the predictor*time interactions are shown only. There were no material changes in significance of main effects. QIC is a relative, ‘lower is better’ measure of goodness of fit. Bold indicates statistical significance. W χ^2^: Wald Chi-square, Β: Beta parameter, SE: Standard Error, CI: Confidence Interval.

## Data Availability

Data is available upon request.

## Supplementary Materials

The following are available online at https://www.mdpi.com/article/10.3390/nu13092911/s1, Table S1: Weighted baseline participant characteristics for the total, included and excluded samples; Table S2: Means with 95% confidence intervals for weight and BMI at each timepoint using complete cases; Table S3: Mean difference in weight/BMI with 95% confidence intervals between timepoints using complete cases; Table S4: Univariate unadjusted and adjusted models for each predictor variable and change in self-reported weight and BMI; Table S5: Unweighted and weighted proportions of underweight, normal weight, overweight and obesity at baseline (May–June 2020), 3 months (August–September 2020) and 6 months follow-up (November–December 2020) using complete cases; Table S6: Univariate unadjusted and adjusted models for each predictor variable and change in self-reported weight and BMI using complete cases; Table S7: Full GEE model containing all predictor variables and the full GEE model containing all predictor variables and significant predictor*time interactions from univariate models adjusted for time using complete cases; Table S8: Univariate unadjusted and adjusted models for each predictor variable and change in self-reported weight as a binary outcome using complete cases; Table S9: Full GEE model containing all predictor variables and the full GEE model containing all predictor variables and significant predictor*time interactions from univariate models adjusted for time, for a change in self-reported weight as a binary outcome using complete cases; Table S10: Univariate unadjusted and adjusted models for each predictor variable and change in self-reported BMI as a binary outcome using complete cases; Table S11: Full GEE model containing all predictor variables and the full GEE model containing all predictor variables and significant predictor*time interactions from univariate models adjusted for time, for a change in self-reported BMI as a binary outcome using complete cases.
